# Improving Braden Scale Documentation for Pressure-Injury Risk Assessment: A Closed-Loop Clinical Audit at Dongola Specialized Hospital

**DOI:** 10.7759/cureus.102455

**Published:** 2026-01-28

**Authors:** Rowa Adil Mohamed Elharadallo, Suzan Mohammed Eltayeb Eltahir, Mohammed Ali Mohammed Ali, Dania Akasha, Ahmed Alsiddig Ebraheem, Raghad Izzeldin Mohamed Elsayed, Fakher Aldeen Raft Fakher Aldeen Noman, Akram Mohamed Yagoub Elfaki, Shaza Awad Ali Badawi, Durrah Adil Sayed Ali, Khulood Khalid Suliman Khalid, Hibatallah Mohammed Ali Abass, Doha Abdelbage Suliman Abdelkareem, Reem Saifeldin Osman, Mohamed Elshafie, Hadeel Ahmed Ramadan Dawod, Ashraqat Abduljawad, Ehab Taha Mohammed Abdalla, Arwa Faisal Mohamed Abdalla, Abdulrahman Abbas Yusuf Mohammed

**Affiliations:** 1 Pediatric Medicine, King Saud University, Riyadh, SAU; 2 Internal Medicine, Sudan Medical Specialization Board, Khartoum, SDN; 3 Internal Medicine, Dongola Specialized Hospital, Dongola, SDN; 4 Internal Medicine, Hamad Medical Corporation, Doha, QAT; 5 Emergency Medicine, Dongola Specialized Hospital, Dongola, SDN; 6 Pediatric Medicine, Dongola Specialized Hospital, Dongola, SDN; 7 Surgery, Dongola Specialized Hospital, Dongola, SDN; 8 Internal Medicine, Yanbu General Hospital, Yanbu, SAU; 9 Emergency Medicine, Bashair University Hospital, Khartoum, SDN; 10 Emergency Medicine, Alhasaheisa Teaching Hospital, Gazira, SDN; 11 Neonatology, Maternity and Children Hospital, Najran, SAU; 12 General Practice, SouthDoc Services Ltd, Cork City, IRL; 13 Internal Medicine, Port Sudan Teaching Hospital, Port Sudan, SDN; 14 Internal Medicine, University of Gezira, Wad Madani, SDN

**Keywords:** braden scale, clinical audit, documentation, pressure injury, quality improvement, risk assessment

## Abstract

Background: Pressure injuries remain a significant and largely preventable cause of morbidity among hospitalized patients. Accurate and complete risk assessment using validated tools such as the Braden Scale is fundamental to effective prevention; however, documentation practices are often inconsistent, particularly in resource-limited settings.

Objective: To evaluate the completeness of Braden Scale documentation in the Internal Medicine Department at Dongola Specialized Hospital and to assess the impact of a targeted quality-improvement intervention through a closed-loop clinical audit.

Methods: A prospective closed-loop clinical audit was conducted over two cycles, each reviewing 51 patient records. Baseline documentation practices (Cycle 1) were assessed against predefined standards derived from international pressure-injury prevention guidelines. Following a structured two-month intervention comprising staff education, reinforcement of documentation expectations, and introduction of a standardized Braden documentation form, a second audit cycle (Cycle 2) was performed using identical criteria. Documentation completeness was analyzed using descriptive statistics, and differences between cycles were assessed using chi-square tests.

Results: At baseline, none of the Braden subscale scores, total scores, risk levels, or preventive actions were documented. Following the intervention, documentation of all six Braden subscales improved to near-universal completion (96.4-100%). Documentation of total Braden score, risk level, and preventive actions increased to 89.1%, 83.6%, and 89.1%, respectively (all p < 0.001). Significant improvements were also observed in consultant identification and bed number documentation. Conversely, completion rates for certain demographic fields, particularly hospital number and admission date, declined in Cycle 2.

Conclusion: This audit demonstrates that targeted education and standardized documentation tools can lead to substantial and statistically significant improvements in Braden Scale documentation. While clinical risk assessment practices improved markedly, persistent gaps in administrative documentation highlight the need for sustained monitoring and system-level reinforcement. Closed-loop clinical audits represent an effective strategy for strengthening pressure-injury risk assessment practices in hospital settings.

## Introduction

Pressure injuries continue to pose a major challenge to healthcare systems worldwide and remain a significant, yet largely avoidable, source of morbidity and mortality among hospitalized patients. Their occurrence is closely linked to prolonged inpatient stays, greater healthcare costs, and considerable physical and psychological distress for affected individuals. Despite advances in clinical practice, a recent global systematic review including over 2.5 million adult inpatients reported a pooled prevalence of 12.8%, revealing persistent regional disparities and demonstrating that pressure injuries remain a substantial clinical burden across diverse healthcare environments [1]. Such findings highlight the need for reliable screening and early intervention, particularly in settings where preventive practices may be inconsistently applied.

To mitigate the burden of pressure injuries, international bodies have developed robust guidelines emphasizing systematic risk assessment and timely implementation of preventive strategies. The 2019 International Guideline issued jointly by the European Pressure Ulcer Advisory Panel (EPUAP), the National Pressure Injury Advisory Panel (NPIAP), and the Pan Pacific Pressure Injury Alliance (PPPIA) outlines risk assessment as a fundamental component of prevention programs and recommends the routine use of validated scoring tools alongside structured skin inspection and care planning [2]. These recommendations underscore that effective prevention is contingent not only on awareness of risk factors but also on accurate, complete, and consistently recorded clinical assessments.

Among existing tools, the Braden Scale is one of the most extensively validated and widely utilized instruments for predicting pressure injury risk. It evaluates six interrelated dimensions-sensory perception, moisture, activity, mobility, nutrition, and friction/shear-to produce a composite score that guides appropriate preventive measures. Early validation studies demonstrated high reliability and strong predictive accuracy, facilitating its integration into routine care across various hospital departments [3]. More recent evidence reinforces its clinical value: studies comparing Braden subscale scores with objective monitoring of patient movement show that well-completed assessments reflect meaningful physiological patterns, enabling personalized prevention strategies and improving clinical decision-making [4]. Conversely, incomplete, inaccurate, or inconsistent documentation diminishes the clinical utility of the tool and increases the likelihood of preventable pressure injuries.

In this context, clinical audit serves as a key mechanism for assessing adherence to best-practice standards and identifying opportunities for improvement. Quality improvement programs that incorporate structured audits, targeted staff training, and standardized documentation have demonstrated substantial reductions in hospital-acquired pressure injuries. For example, a multidisciplinary initiative implemented in a tertiary cardiac center in Qatar achieved an 83.5% reduction in pressure injury incidence over four years through sustained education, preventive bundles, and continuous monitoring [5]. Such results illustrate the transformative potential of systematic auditing, particularly in resource-constrained settings where workflow interruptions, limited staffing, or inconsistent documentation practices may contribute to gaps in care.

At Dongola Specialized Hospital, preliminary observations suggested variability in how the Braden Scale was applied and documented within the Internal Medicine Department. These inconsistencies raised concerns about the reliability of risk assessment processes and the potential for missed opportunities to implement timely preventive interventions. In response, this clinical audit was undertaken to evaluate current practices, measure compliance with Braden Scale documentation standards, and assess the impact of targeted interventions implemented between two audit cycles. By examining changes in documentation quality, this study aims to strengthen local pressure injury prevention strategies and reinforce the integration of standardized risk assessment into routine inpatient care.

## Materials and methods

Study design

This project was conducted as a prospective, closed-loop clinical audit aimed at evaluating and improving Braden Scale documentation within the Internal Medicine Department of Dongola Specialized Hospital. The audit followed the traditional audit cycle, beginning with a baseline review of existing documentation practices (Cycle 1), followed by implementation of targeted corrective measures, and concluding with a second-cycle reassessment (Cycle 2) using the same standards and data-collection procedures. The overall duration of the audit extended across approximately six months.

Study setting

The audit was carried out in the Internal Medicine ward of Dongola Specialized Hospital, a secondary-level teaching and referral institution where the Braden Scale is routinely used to assess patients’ risk of developing pressure injuries. Documentation of the Braden Scale is expected at the time of admission for all patients who are vulnerable to reduced mobility or impaired skin integrity. The audit was undertaken in response to observed inconsistencies in the completion of Braden assessments and aimed to determine the extent to which routine documentation adhered to institutional expectations.

Audit timeline

The first audit cycle involved a review of patient records to establish baseline documentation performance prior to the introduction of any interventions. After completing Cycle 1, the audit team conducted a structured two-month intervention phase designed to address the shortcomings identified in the initial assessment. Subsequently, Cycle 2 was conducted prospectively over a three-month period, applying the same criteria, assessment structure, and data-collection approach to ensure comparability between the two cycles and to objectively determine the impact of the intervention.

Study population and sample size

The audit reviewed a total of 51 Internal Medicine patient records in the second cycle, corresponding to the available admissions during the designated audit period. All patient files that were expected to include a Braden assessment upon admission were eligible for inclusion. No exclusion criteria were applied, as the intention was to capture an authentic representation of routine documentation practices within the ward and to evaluate the hospital's overall adherence to Braden Scale requirements.

Audit standards and evaluation criteria

Audit standards were developed based on established international pressure injury prevention guidelines and institutional policies governing Braden Scale documentation. Each medical record was examined for the completeness of several core documentation elements, including patient identifiers, admission details, and the clinical diagnosis recorded at presentation. The audit also assessed whether all six Braden subscales--sensory perception, moisture, activity, mobility, nutrition, and friction or shear--had been documented, along with the total Braden score and the assigned risk category. Documentation was further reviewed for evidence of preventive actions taken and for the name or initials of the staff member completing the assessment. A documentation element was considered complete only when all required information within that category was fully and accurately recorded.

Data collection procedures

During Cycle 1, data were extracted retrospectively from physical patient records using a structured checklist specifically designed to promote consistency across reviewers. In Cycle 2, data were collected prospectively using the same checklist and criteria to maintain methodological alignment across cycles. All extracted data were entered into a customized Google Forms tool (Google LLC, Mountain View, CA), which generated a corresponding spreadsheet that served as the primary dataset for analysis.

Intervention between audit cycles

After completing the baseline assessment, the audit team introduced a targeted intervention intended to improve the accuracy and consistency of Braden documentation. Over a two-month period, staff received structured educational sessions that clarified appropriate Braden scoring, emphasized accurate interpretation and documentation, and highlighted errors frequently identified in Cycle 1. Ward leadership reinforced documentation expectations during handovers and daily rounds, and the Braden documentation form was standardized to improve usability and clarity. The intervention was designed to enhance awareness of pressure injury risk assessment and to promote reliable integration of Braden scoring into routine clinical workflows.

Data analysis

Data exported from Google Forms were analyzed using descriptive statistics to summarize the completeness of each documentation item. Each element was coded dichotomously as completed or not completed, and completion percentages for both cycles were calculated. Differences in documentation performance between Cycle 1 and Cycle 2 were quantified as absolute percentage changes. To determine whether improvements were statistically significant, chi-square tests were applied to compare proportions across the two cycles. A significance threshold of p < 0.05 was used for all comparisons, and chi-square values, degrees of freedom, and p-values were reported for each documentation parameter.

Ethical considerations

This clinical audit was conducted in accordance with the ethical and governance requirements of Dongola Specialized Hospital and the Northern State Ministry of Health. As the project involved a review of routinely collected clinical documentation with no direct patient contact or intervention, the requirement for informed patient consent was waived. All data were handled confidentially, and patient identifiers were removed prior to analysis to ensure anonymity.

Formal ethical approval and institutional recognition for this audit were obtained from Dongola Specialized Hospital under IRB Protocol Number: AU0997, as documented in the Clinical Audit Approval and Certificate of Recognition. The audit was officially approved as a quality-improvement initiative aimed at enhancing pressure-ulcer prevention practices through the implementation of the Braden Scale.

## Results

A total of 51 patient records were reviewed in each audit cycle to assess the completeness of Braden Scale documentation in the Internal Medicine Department. The findings revealed substantial improvements in most clinical components of the Braden Scale following the intervention, although some demographic elements demonstrated a decline in completeness between cycles.

In Cycle 1, completion rates for patient identifiers were generally high, with 100% of records documenting the patient's name and age, 97.73% including the admission date, and 88.64% documenting the hospital number. However, in Cycle 2, documentation of patient identifiers declined in several areas, with the patient’s name recorded in 96.36% of files, age in 92.73%, and date in 83.64%. The hospital number showed the greatest reduction, decreasing markedly from 88.64% in Cycle 1 to 27.27% in Cycle 2 (χ² = 38.64, p < 0.000000001). In contrast, documentation of ward/unit assignment and the responsible consultant improved significantly, rising from 61.36% to 92.73% (χ² = 13.95, p < 0.001) and from 18.18% to 83.64% (χ² = 45.35, p < 0.000000001), respectively.

The most notable improvements occurred in the clinical components of the Braden Scale. In Cycle 1, none of the six Braden subscale scores-sensory perception, moisture, activity, mobility, nutrition, or friction/shear-were documented in any of the patient records. Following the intervention, documentation rates increased dramatically in Cycle 2, reaching 100% for sensory perception, moisture, activity, and nutrition; 98.18% for friction/shear; and 96.36% for mobility. All improvements were statistically significant, with χ² values exceeding 94 for all subscale items (p < 0.00000000001). These gains reflect a major shift toward consistent completion of core pressure-injury risk assessment elements.

Similarly, documentation of the total Braden score, assigned risk level, and actions taken or preventive measures-none of which were completed in Cycle 1-improved significantly in Cycle 2, with completion rates of 89.09%, 83.64%, and 89.09%, respectively. These improvements were also highly statistically significant (all p < 0.00000001). Documentation of the assessor’s name or initials increased from 0% to 81.82% (χ² = 71.40, p < 0.00000000000029), indicating strengthened accountability within the assessment process.

Overall, the results demonstrate that the intervention led to substantial and statistically significant improvements in nearly all clinical and risk-related components of the Braden Scale assessment. However, the decline observed in certain demographic fields, particularly hospital number, date, and age, highlights the need for reinforced training and systematic monitoring to ensure that both demographic and clinical documentation standards are consistently met (Table 1).

**Table 1:** Completeness of Braden Scale documentation across audit cycles at Dongola specialized hospital (Cycle 1 vs Cycle 2). This table presents the frequencies and percentages of completed documentation items for each required component of the Braden Scale assessment in Cycle 1 (n = 51) and Cycle 2 (n = 51). Chi-square (χ²) tests were used to compare proportions between cycles to determine whether changes in documentation completeness were statistically significant. A p-value of <0.05 was considered statistically significant.

Item	Cycle 1	Cycle 2	Chi-square (χ²)	p-value
Patient name	51 (100.0%)	49 (96.36%)	2.04	0.153
Patient age	51 (100.0%)	47 (92.73%)	4.16	0.041
Ward unit	31 (61.36%)	47 (92.73%)	13.95	<0.001
Consultant	9 (18.18%)	43 (83.64%)	45.35	<0.000000001
Hospital number	45 (88.64%)	14 (27.27%)	38.64	<0.000000001
Bed number	1 (2.27%)	37 (72.73%)	54.36	<0.000000000001
Diagnosis	42 (81.82%)	45 (89.09%)	0.70	0.402
Date	50 (97.73%)	43 (83.64%)	5.97	0.015
Sensory score	0 (0.0%)	51 (100.0%)	102.00	<0.000000000000000000000056
Moisture	0 (0.0%)	51 (100.0%)	102.00	<0.000000000000000000000056
Activity	0 (0.0%)	51 (100.0%)	102.00	<0.000000000000000000000056
Mobility	0 (0.0%)	49 (96.36%)	94.30	<0.0000000000000000000027
Nutrition	0 (0.0%)	51 (100.0%)	102.00	<0.000000000000000000000056
Friction/shear	0 (0.0%)	50 (98.18%)	98.08	<0.0000000000000000000040
Total score	0 (0.0%)	45 (89.09%)	80.53	<0.0000000000000029
Risk level	0 (0.0%)	43 (83.64%)	74.34	<0.000000000000066
Action taken/notes	0 (0.0%)	45 (89.09%)	80.53	<0.0000000000000029
Assessor	0 (0.0%)	42 (81.82%)	71.40	<0.00000000000029

## Discussion

This closed-loop clinical audit demonstrated that a structured quality-improvement intervention, combining focused staff education with the implementation of a standardized Braden Scale documentation form, resulted in clear and statistically significant improvements in the completeness of pressure-injury risk documentation. At baseline, essential patient identifiers and administrative fields were inconsistently recorded, and none of the Braden subscales, total scores, or preventive measures were documented. This pattern of incomplete documentation is consistent with international evidence highlighting substantial discrepancies between recorded pressure-injury risk assessments and actual bedside findings, with routine nursing documentation frequently underrepresenting both patient risk status and preventive practices [6].

Following the intervention, documentation completeness improved markedly across nearly all assessed domains, reflecting the immediate effectiveness of targeted education and simplified documentation processes. Improvements were particularly notable within the clinical components of the Braden Scale, where the transition from complete non-documentation in Cycle 1 to universal or near-universal completion in Cycle 2 represents a substantial enhancement in risk-assessment practice. Similar outcomes have been reported in previous studies, including Gunningberg’s evaluation of a nurse-training programme in Sweden, where structured education led to improved adherence to preventive routines and more accurate pressure-injury documentation [7]. The consistency between these findings and the broader literature suggests that even in resource-limited settings, focused training supported by clear documentation tools can rapidly improve compliance with best practice.

The clinical relevance of these improvements is supported by strong evidence underpinning the Braden Scale itself. A systematic review and meta-analysis by Huang et al. confirmed the predictive validity of the Braden Scale in identifying hospitalized adults at risk of pressure injuries [8]. More recent validation work by Kennerly and colleagues further emphasizes the importance of complete subscale documentation, demonstrating that activity and mobility scores correlate significantly with objective, sensor-based movement data [4]. These findings indicate that comprehensive Braden documentation reflects meaningful clinical assessment rather than a purely administrative exercise. Accordingly, the improvements observed in this audit represent closer alignment with evidence-based risk-assessment standards.

Quality-improvement literature further supports the role of structured documentation and bundled prevention strategies in reducing pressure-injury burden. A hospital-wide initiative in Qatar achieved an over 80% reduction in pressure-injury incidence through routine staff training, standardized assessment tools, and continuous monitoring [5]. Although the present audit did not directly evaluate clinical outcomes, the principles underlying these successful interventions closely mirror those applied in this study, reinforcing the potential downstream impact of improved documentation on patient safety.

Despite the overall progress achieved, some documentation gaps persisted, particularly in administrative elements such as hospital numbers and bed identifiers. Similar challenges have been reported internationally. A recent Swedish review found that only 2.1% of care plans and 4.7% of nursing notes contained complete pressure-injury risk documentation, highlighting persistent global deficiencies in documentation compliance despite established guidelines [9,10]. These findings suggest that improvements in clinical assessment documentation do not automatically extend to administrative fields and may require additional system-level reinforcement.

Evidence from audit-and-feedback research underscores the importance of ongoing monitoring to sustain improvement. A longitudinal, multicenter study in nursing homes demonstrated that regular audits, combined with feedback and preventive-care reminders, significantly improved adherence to repositioning protocols and increased the use of pressure-relieving surfaces, ultimately reducing pressure-injury prevalence over time [11]. Implementation science further indicates that multifaceted strategies--integrating education, standardized documentation templates, multidisciplinary engagement, and continuous evaluation--are more effective than isolated interventions in sustaining quality improvement [12,13]. High-quality documentation also carries broader implications, supporting clinical decision-making, improving communication, and strengthening medico-legal accountability [14]. A systematic review by Bunting and de Klerk reinforced that programmes incorporating education, structured forms, audit cycles, and feedback consistently improve documentation adherence in hospital settings [15].

Several limitations should be considered when interpreting these findings. This audit was conducted within a single institution, potentially limiting generalizability to other healthcare settings with different workflows or resource constraints. The evaluation focused exclusively on documentation completeness rather than patient-centered clinical outcomes; therefore, it was not possible to assess whether improved Braden documentation resulted in a reduction in pressure-injury incidence or severity. Data collection relied on retrospective review of paper-based medical records, which may be subject to missing data, filing inconsistencies, and documentation bias. In addition, the post-intervention assessment was performed shortly after implementation, and the long-term sustainability of the observed improvements remains uncertain.

In summary, this audit demonstrates that targeted, low-cost quality-improvement interventions can rapidly and meaningfully enhance the completeness of pressure-injury risk documentation. While limited by its scope and focus on documentation outcomes, the findings highlight the effectiveness of closed-loop audits in strengthening adherence to risk-assessment standards. Future work should incorporate longer follow-up periods and patient-level outcome measures to determine whether these documentation improvements translate into sustained reductions in pressure-injury incidence and improvements in patient care quality.

## Conclusions

This closed-loop clinical audit demonstrated that targeted quality-improvement measures, specifically staff education and the introduction of a standardized Braden Scale documentation tool, led to substantial and statistically significant enhancements in the completeness of pressure-injury risk assessment at Dongola Specialized Hospital. The transition from entirely absent Braden documentation in Cycle 1 to near-universal completion of all subscales, total scores, risk levels, and preventive notes in Cycle 2 reflects a meaningful shift toward more consistent and evidence-based assessment practices. These improvements provide a critical foundation for early risk identification and more effective prevention strategies. However, declines in certain demographic and administrative fields highlight the need for sustained monitoring, continued staff reinforcement, and additional workflow optimization to ensure that all components of the assessment process are reliably completed. Although this audit did not measure patient outcomes, the marked improvement in documentation completeness underscores the value of closed-loop auditing in strengthening clinical practice. Future work should examine the long-term sustainability of these improvements and evaluate whether enhanced documentation translates into measurable reductions in pressure-injury incidence.
